# Predicting the start week of respiratory syncytial virus outbreaks using real time weather variables

**DOI:** 10.1186/1472-6947-10-68

**Published:** 2010-11-02

**Authors:** Nephi A Walton, Mollie R Poynton, Per H Gesteland, Chris Maloney, Catherine Staes, Julio C Facelli

**Affiliations:** 1 Department of Biomedical Informatics, University of Utah, Salt Lake City, Utah, USA; 2 College of Nursing, University of Utah, Salt Lake City, Utah, USA; 3 Department of Pediatrics, University of Utah, Salt Lake City, Utah, USA; 4 Center for High Performance Computing, University of Utah, Salt Lake City, Utah, USA

## Abstract

**Background:**

Respiratory Syncytial Virus (RSV), a major cause of bronchiolitis, has a large impact on the census of pediatric hospitals during outbreak seasons. Reliable prediction of the week these outbreaks will start, based on readily available data, could help pediatric hospitals better prepare for large outbreaks.

**Methods:**

Naïve Bayes (NB) classifier models were constructed using weather data from 1985-2008 considering only variables that are available in real time and that could be used to forecast the week in which an RSV outbreak will occur in Salt Lake County, Utah. Outbreak start dates were determined by a panel of experts using 32,509 records with ICD-9 coded RSV and bronchiolitis diagnoses from Intermountain Healthcare hospitals and clinics for the RSV seasons from 1985 to 2008.

**Results:**

NB models predicted RSV outbreaks up to 3 weeks in advance with an estimated sensitivity of up to 67% and estimated specificities as high as 94% to 100%. Temperature and wind speed were the best overall predictors, but other weather variables also showed relevance depending on how far in advance the predictions were made. The weather conditions predictive of an RSV outbreak in our study were similar to those that lead to temperature inversions in the Salt Lake Valley.

**Conclusions:**

We demonstrate that Naïve Bayes (NB) classifier models based on weather data available in real time have the potential to be used as effective predictive models. These models may be able to predict the week that an RSV outbreak will occur with clinical relevance. Their clinical usefulness will be field tested during the next five years.

## Background

Bronchiolitis is a major cause of hospital admissions during the winter and can cause severe hospital overcrowding. Respiratory syncytial virus (RSV) is a respiratory virus that can cause severe infection in infants and young children and is the leading cause of bronchiolitis in children under one year of age in the United States [1-4]. RSV outbreaks cause a significant increase in hospital admissions during the winter season [2]. The ability to predict the start date of an RSV outbreak using readily available data may allow for the implementation of management strategies in a more timely, effective, and efficient fashion. Some possible improvements may include: improved staff scheduling, improved rescheduling of elective procedures, anticipatory resource utilization and mobilization of respiratory supplies, improved timing of control measures, and improved timing for restricting visitation and grouping patients [5,6].

RSV has a characteristic biennial outbreak pattern with alternating low peak and high peak seasons [2]. While exceptions of this biennial pattern have been reported in the literature [7-11] we observed the normal biennial pattern in all the data used in this study. It has been shown using mathematical models that this pattern of high peak and low peak outbreaks can be explained by the variation in the number of susceptible individuals in the population, which is considerably diminished following a large outbreak [1,4,12]. The 'high peak' seasons tend to occur earlier in the year, have a higher peak, and a shorter duration. In contrast, the 'low peak' seasons occur later, have lower peaks, and a longer duration [4]. If, as suggested by the mathematical models, the different patterns observed for high and low seasons were due to the size of the susceptible population, it also would be expected that on a low outbreak season there would be a greater lag between outbreak stimulus and the exponential growth of confirmed RSV cases. The inference is that it takes the outbreak longer to infect a large number of individuals given the level of immunity already existing in the population due to large outbreaks during the previous year. Therefore, it is important to develop independent models for high peak and low peak years.

Although existent mathematical models describe RSV outbreaks, the prediction of outbreak start dates remains an unsolved problem [1,3,4,12]. Current methods of identifying outbreak start date are all based on retrospective data. To our knowledge, there are no studies published in the biomedical literature that attempt to predict the start week of an RSV outbreak using variables that can be acquired in real time and that are appropriate for inclusion in a forecast model that can be used in a clinical setting. Among these variables, weather-related variables (such as temperature, humidity, and precipitation) have great potential to be predictive because many studies have correlated them with RSV outbreaks [13-16]. To construct a predictive model for RSV outbreaks, one must consider the small training set resulting from the lack of long term epidemiology records and the need to separate the data between low and high peak seasons. Therefore, the objective of our investigation was to explore the feasibility of using weather variables with Naïve Bayes (NB) classifiers to predict RSV outbreaks and the concomitant increase in RSV-related admissions to a pediatric hospital.

## Methods

### Weather Data

Weather data from 1985 to 2008 were obtained from the National Oceanic and Atmospheric Administration (NOAA) National Climatic Data Center (http://cdo.ncdc.noaa.gov/CDO/cdo). Data from the Salt Lake International Airport weather station were used to represent weather for the entire Salt Lake County region.

### RSV Diagnosis

Data representing the diagnosis of RSV were obtained from the Intermountain Healthcare Enterprise Data Warehouse (EDW). We selected records for patients that reside in Salt Lake County, Utah that meet the following criteria: 1) laboratory confirmation of RSV by viral culture or direct fluorescent antibody (DFA) or/and polymerase chain reaction (PCR); or 2) a discharge diagnosis coded with a bronchiolitis or RSV-related ICD-9 code, including: 466 acute bronchitis and bronchiolitis; 466.1 acute bronchiolitis; 466.11 acute bronchiolitis due to respiratory syncytial virus (RSV); and 466.19 acute bronchiolitis due to other infectious organism. We selected patients from of all ages meeting the selection criteria given above; the age of the patients included in the study ranges from newborn (0 days) to age 95, with the median age of 6 months.

For the purpose of this study, an RSV season was defined as September 20^th ^to July 15^th ^of the following year. We used the diagnostic ICD-9 codes to select records from 1985 through 2008, providing data for 12 high- and 11 low-peak seasons. We used the laboratory criteria defined above to select records from 2002 through 2008. Viral testing laboratory data were not available prior to 2001; viral testing was routinely performed on patients seen at PCMC after 2003. Between August 24, 2004 and June 26, 2008, RSV-related laboratory results and ICD-9 codes for non-specific bronchiolitis were highly correlated (Kendall tau correlation statistic = 0.78; see also in the Additional file 1 the superposition graph of RSV and ICD9 cases for this period). This indicates that ICD-9 codes are a reasonable proxy for positive laboratory tests. While some literature results [17] shows that outbreaks of other respiratory pathogens like HMPV, dual out breaks, etc. may invalidate this assumption, the ICD9 signal observed in our data does not present any evidence of these issues, giving confidence that our assumption to use ICD9 codes as a proxy for RSV cases is valid for our study.

### Determination of the start Week of the Outbreak

**Figure 1:**
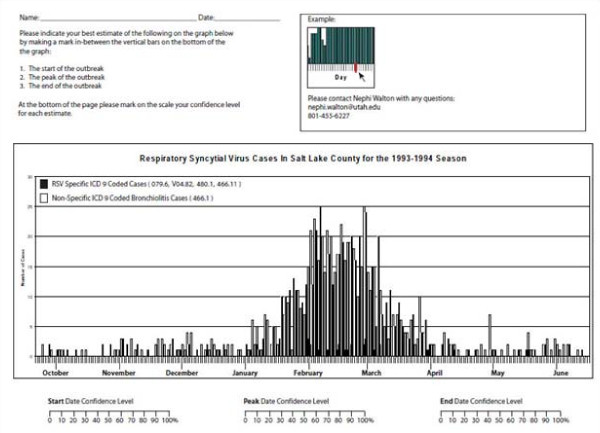
**Copy of one page of the survey given to the expert panel to determine the start dates of the RSV outbreaks**. A similar page was provided for each of the years considered in this study.

Due to limitations of our data, it was first necessary to determine a reference standard for the start date of the outbreak to train and test our models. Currently, epidemiologists with the Center for Disease Control (CDC) use percent positive lab tests [18] and infectious disease specialists with Primary Children's Medical Center in Salt Lake City, Utah use the number of laboratory confirmed cases in a seven day window to determine the presence of an outbreak. Unfortunately, we were unable to use these criteria based on laboratory data to define the outbreaks because viral testing for RSV was not routinely available prior to 2001. In the absence of laboratory data, we used domain expert opinion in combination with available information from ICD9 codes to establish the reference standard for presence of outbreak [19-21]. Expert opinion was obtained by creating graphs of the number of patient records with ICD-9 codes for RSV and bronchiolitis over time for each season considered in this study (Figure 1). Each graph showed both the number of non-specific bronchiolitis ICD-9 coded cases for each day along with the number of RSV specific ICD-9 codes. RSV and bronchiolitis activity in the graphs was shown from mid September to mid June encompassing the entire RSV season for the seasons from 1985 to 2008. Ten infectious disease experts from the University of Utah Department of Pediatrics were asked to independently assess the date at which the outbreak started for each season. The outbreak start week was then chosen based on the Sunday of the week a majority of the physicians identified as the outbreak starting date.

### Weather Variable Selection

To select weather variables to be considered for the prediction model, we reviewed the literature and the availability and utility of variables identified. We identified publications that reported either a positive or negative relationship between RSV activity and weather. Among the 40 publications, the following weather variables either correlated or did not correlate with RSV outbreaks: humidity (16:12), dew point (4:5), temperature (26:9), wind speed (0:3), wind chill (1:0), atmospheric pressure (3:6), precipitation (8:8), and UV light exposure (7:8) [22]. We discarded UV light as a potential variable because there was insufficient data available for inclusion in our prediction model. We discarded dew point and wind chill because these variables can be derived from other variables already included in the model (temperature and humidity, and wind speed and temperature, respectively). The remaining weather variables were retained to be considered in our models. We used two measurements to represent humidity (daily minimum relative humidity and daily maximum relative humidity) and three measurements to represent temperature (mean daily temperature, minimum daily temperature, and maximum daily temperature). Precipitation, atmospheric pressure, and wind speed were each represented by a single daily measurement. Attempts to use feature selection methods to further reduce the number of variables in our models were unsuccessful. Therefore, the following eight measurements (variables) were included in our model: daily minimum relative humidity, daily maximum relative humidity, mean daily temperature, minimum daily temperature, maximum daily temperature, total daily precipitation, average daily atmospheric pressure, and average daily wind speed.

### Naive Bayes Classifier

**Figure 2:**
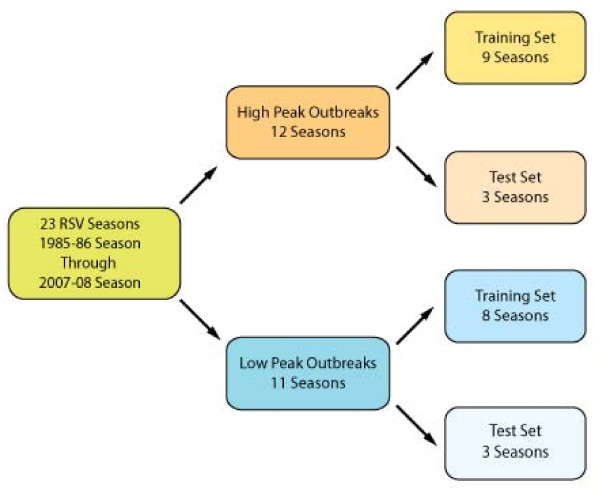
**Schema showing how the 23 years of data available for this study were segregated into high and low peak seasons and training and test data sets**. An RSV season was defined as September 20th to July 15th of the following year.

Among the 23 seasons available for analysis, there were 12 high- and 11-low peak outbreak seasons (Figure 2). The early seasons were used for training the models and the three latest seasons were used for testing the high- and low-peak models (Figure 2). We induced Naive Bayes (NB) models for every possible combination of the eight selected variables; thus, we induced a total of 255 unique NB classifiers. The exhaustive building and comparison of all possible models was motivated by unsuccessful attempts to use variable selection algorithms with our current data. The Naïve Bayes classifier models were generated using Statistica Data Miner version 9 (Stat Soft, Inc., Tulsa, OK, U.S.A.).

**Figure 3:**
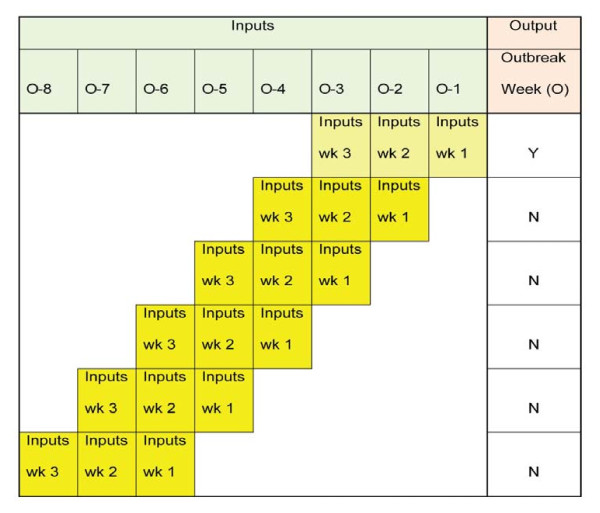
**Visual representation of how the input data for the NB classifier are structured**.

The NB classifier was trained with a series of values for daily weather variables and a flag indicating whether, according to the determination of the expert panel, an outbreak occurred in the week following the last Sunday in the data set. As shown in Figure 3, models were run with three weeks of data as input (i.e. for each three week period, each weather variable has 21 values, one value for every day of each week). Separate classifiers were built for predicting the outbreak in the same week, one week in advance of the outbreak, two weeks in advance of the outbreak, and three weeks in advance of the outbreak, respectively. To predict the outbreak in advance, a sliding window was used. The output remained the same but the input window would slide back one week, overlapping the data used in the previous prediction by two weeks. This process was repeated again as the prediction week was moved forward by sliding the window back one week each time (see Figure 3). In the training sets for each week in which an outbreak occurred, there were six weeks of data included prior to when the outbreak occurred. These weeks were set to a negative flag value when no outbreak occurred. For the high peak years, there were 9 'outbreak' weeks and 54 'no outbreak' weeks in the training set and three 'outbreak' weeks and 18 'no outbreak' weeks in the test set. Similarly, for the low peak years, there were 8 'outbreak' weeks and 48 'no outbreak' weeks in the training set, and three 'outbreak' weeks and 18 'no outbreak' weeks in the test set. Unique NB classifiers were induced for every possible combination of variables in both high and low peak seasons for 0, 1, 2, and 3 weeks in advance of the outbreak, generating a total of 2040 NB models. The comprehensive list of the weather variables used in each of the models considered, along with the sensitivity and specificity in both training and test sets for both high peak and low peak seasons, are presented in the Additional file 2.

Institutional Review Board approval to perform this study was obtained from Intermountain Healthcare and the University of Utah.

## Results

### Model performance

**Figure 4:**
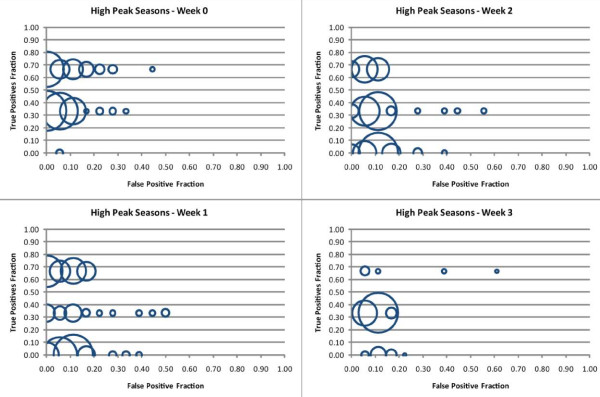
**The true positive fraction (y-axis) is graphed against false positive fraction (x-axis) for NB classifier models for high peak seasons and for every possible variable combination (n = 255) of the weather variables used in this study**. The size of each circle represents the number of NB classifier models that obtained the given accuracy. Each model predicts an RSV outbreak the Sunday preceding the outbreak (Week 0), one week in advance (Week 1), two weeks in advance (Week 2), and three weeks in advance (Week 3).

For the high peak seasons, the NB classifier models predicting the outbreak in the same week achieved a sensitivity of 67% with specificities of up to 100% on the test set. This accuracy was achieved by 51 of the 255 different models that can be constructed using all the possible combinations of the weather variables tested in this study. It is important to note however that the reduced test set of three years means that sensitivity and specificity can only have the values of 0, 1/3, 2/3 and 1. The best models predicting the outbreak one week in advance achieved the same accuracy as those predicting the outbreak in the same week, but only 35 of the 255 variable combinations achieved this level of performance. Only nine models predicting the outbreak two weeks in advance achieved the same accuracy. Finally, the seven best models predicting the outbreak three weeks in advance reached the same sensitivity, 67%, but had a lower maximum specificity of 94%. Figure 4 depicts the true positive fraction and false positive fraction for the test set for all possible NB models using the 255 combinations of weather variables to predict high peak outbreaks in the week of the outbreak (week 0), one week in advance (week 1), two weeks in advance (week 2) and three weeks in advance (week 3).

**Figure 5:**
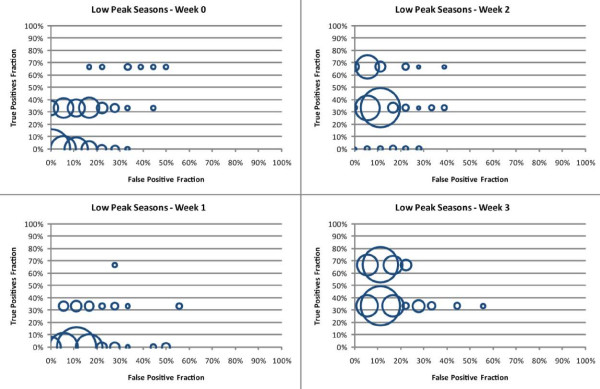
**The true positive fraction (y-axis) is graphed against false positive fraction (x-axis) for NB classifier models for low peak seasons and for every possible variable combination (n = 255) of the weather variables used in this study**. The size of each circle represents the number of NB classifier models that obtained the given accuracy. Each model predicts an RSV outbreak the Sunday preceding the outbreak (Week 0), one week in advance (Week 1), two weeks in advance (Week 2), and three weeks in advance (Week 3).

For the low peak seasons, the NB classifier models predicting the outbreak in the same week achieved a sensitivity of 67% with a specificity of up to 83%. This accuracy was achieved by only one of the 255 possible models, with most of the other models (see Figure 5) achieving results no better than chance (100% combined sensitivity and specificity on the test set). Models predicting the outbreak one week in advance produced similar results, with only one achieving the same sensitivity but with a decreased specificity of 72%. Most other models also performed at or below chance (see Figure 5). Overall predictive accuracy increased significantly for models attempting to predict the outbreak two or three weeks in advance. The six best models predicting the outbreak two weeks in advance achieved a sensitivity of 67% with a specificity of 100% and the 32 best models predicting the outbreak three weeks in advance achieved a sensitivity of 67% and a specificity of 94%. Figure 5 depicts the true positive fraction and false positive fraction for the test set for all possible NB models using the 255 combinations of weather variables to predict low peak outbreaks in the week of the outbreak (week 0), one week in advance (week 1), two weeks in advance (week 2) and three weeks in advance (week 3).

As discussed above, in most cases there was no single model that can be clearly considered the best for predicting the start of an outbreak one, two or three weeks in advance. Inspection of the Tables in the Additional file 2 as well as Figures 4 and 5 show that numerous models can be considered equally appropriate, depending on the selection criteria (i.e. sensitivity, specificity, different combinations of them, performance on the test set or the training set, etc.) Given the limited number of outbreaks in our testing data, this finding is not surprising. A larger set of testing data is necessary to adequately describe performance.

### Individual variable performance

The relative importance of a single weather variable contributing to the best performing models was defined as the percentage of times a variable was included in the best performing models for each type of prediction (Table 1). Best performing models were defined as those with a sensitivity of at least 67% and a specificity of at least 94%. For high peak seasons, wind speed is the variable most consistently encountered in the best models making predictions for the week of the outbreak and one and two weeks in advance, but it is less represented in the best models for predicting three weeks in advance of the outbreak. The three temperature (minimum, average and maximum) variables are similarly represented in the best models that predict for the week of the outbreak and one week in advance, however, minimum temperature is more frequently included in the models that best predict the outbreak two and three weeks in advance. As shown in Table 1, three variables (atmospheric pressure, maximum and minimum relative humidity) were found in the best models that predict for the week of the outbreak and one week in advance, but were less likely to be found in models that predict outbreaks two and three weeks in advance. Precipitation was not present in any of the top performing models for high peak outbreak seasons.

**Table 1:** Percentage of models that include each variable in the highest performance models, based on achieving a minimum sensitivity of 67% and minimum specificity of 94%, for each advanced prediction for high peak seasons.

	Average Daily Pressure	Average Daily Wind Speed	Minimum Humidity	Maximum Humidity	Daily Precipitation	Minimum Temperature	Maximum Temperature	Average Daily Temperature
**Week 0**	47%	94%	57%	51%	0%	55%	63%	55%
**Week 1**	44%	86%	58%	46%	0%	54%	64%	64%
**Week 2**	24%	47%	29%	74%	0%	71%	68%	71%
**Week 3**	14%	29%	0%	0%	0%	100%	57%	71%

For low peak seasons, the analysis of the weather variables included only the models that can reasonably predict an outbreak. The predictions for the week of the outbreak and one week in advance of the outbreak are probably not significant since these models have only one variable combination that achieves the top performance and they both perform much worse than any other model considered here. For top performing models in low peak seasons, wind speed appears in over 50% of the best models for predictions two weeks in advance and then drops to less than 20% for predictions three weeks in advance. The minimum temperature appears in almost 60% of the predictive models for predictions two weeks and three weeks in advance. Similar to high peak seasons, precipitation was not present in any of the top performing models for low peak seasons. Atmospheric pressure appears in 51% of the models predicting 2 weeks in advance and drops to 22% of the models predicting the outbreak three weeks in advance. Maximum relative humidity appears in 51% of models predicting two weeks in advance while minimum relative humidity only appears in 2% of models two weeks out, both have identical representation in predictions three weeks out, appearing only in 22% of the top performing models.

## Discussion

Overall, the input variable temperature, expressed as maximum, average or minimum, consistently appears in the best predictive models, with one of the three temperature variables appearing in over 90% of the best performing models for any prediction attempted. The importance of temperature as a predictive variable increases the further in advance the predictions are made. The atmospheric pressure appears to be an important factor for predicting the outbreak in its week and one week in advance, but it is encountered less frequently in predictions models for two and three weeks in advance. These results show that the variables most commonly encountered in the best models for predicting RSV outbreaks are similar to those associated with the development of temperature inversions in the Salt Lake Valley [23]. It is important to note that these inversions are always associated with a severe increase in levels of air pollutants that have been consistently correlated with respiratory health issues [24-26].

To further our investigation, we attempted to use air pollution indicators as predictive factors for RSV outbreaks. Several attempts were performed to develop NB models using air pollution variables (e.g., PM 10, and concentration of CO and SO_2_) without success. Unfortunately, the data available for concentrations of PM 2.5, an air pollution indicator that has shown some association with bronchiolitis [27-29], is not sufficient to appropriately train a NB classifier. Analysis of only nine years of PM 2.5 data suggested that the concentration of these particles may have predictive value to forecast RSV outbreaks, but definite answers must wait until sufficient years of retrospective PM 2.5 data becomes available.

According to our literature review, wind speed has not been reported as a good predictor for RSV outbreaks. In contrast, wind speed was predictive in our analysis. The unique geography of the Salt Lake Valley contributing to the common occurrence of inversions in winter may account for this discrepancy, as wind (or lack thereof) plays a vital role in the creation of inversions.

The difference in findings for high and low peak outbreak years agree well with what it is expected if RSV outbreak biannual patterns are a result of changes in the susceptible population. In low peak years, the number of susceptible individuals is lower than in high peak years; therefore, it is expected that even if the meteorological conditions exist to start an outbreak, the time for the outbreak to spread will be longer due to herd immunity. The speed of transmission is slower when more of the population exhibits immunity. This observation agrees very well with our relative lack of success in predicting outbreaks during the week the outbreak occurred and one week in advance during low peak seasons. In contrast, during high peak years, the number of cases quickly ramp up once the appropriate meteorological conditions exist to start the outbreak, leading to good predictive power for these short-term predictions.

Our study has limitations. Data limitations allowed for only rough performance estimates for unique models. Because of the limited amount of seasons on which this model was tested, it is possible that large changes in sensitivity and specificity could be a reflection of limited data rather than actual model performance. The question of which model to implement in practical applications remains unanswered, and will depend upon refined performance estimates as data availability increases or the desire to select models with increased sensitivity or specificity to meet the operational needs of the hospital. Finally, we acknowledge that serious outbreaks from other viral pathogens can interfere with the RSV outbreaks making the prediction of a weather based model less reliable. Despite these limitations, the results validate our decision to use different models for high and low peak seasons and are consistent with the existing models to explain high and low peak seasons based on the size of the susceptible population. To address the limitations we identified, we will evaluate prediction models prospectively in the Salt Lake County region for the next five years.

Use of the results presented here in other geographic locations would require NB training with local weather data to account for the changing characteristics of RSV outbreaks in different regions with different climates and varying geographic features. However, it is likely that the same climate variables and methods could be used to build a predictive model in other locations, particularly if they have similar climates and geography to the Salt Lake County area.

## Conclusion

Weather-related measurements available in real time have the potential to predict RSV outbreaks. Our results are consistent with previous studies that indicated low peak and high peak outbreak seasons have different population dynamics, which most likely would lead to a lag between stimulus and event for low peak outbreak seasons. In our study, it appears that weather conditions that lead to outbreaks may be conditions that also lead to the establishment of a temperature inversion in the Salt Lake Valley, which in turn creates a condition of more polluted air known to impact respiratory health. In the future, NB prediction models should include pollution measurements as inputs, and validation should be performed prospectively.

## Competing interests

The authors declare that they have no competing interests.

## Authors' contributions

NW designed and developed the methodology, performed the data preparation and analyses, and drafted the manuscript. MRP contributed to the design of the study, analysis of results and manuscript preparation and revision. PG contributed to the design of the project, the acquisition of data and the critical review and revision of the manuscript. CM contributed to the design, data acquisition, interpretation of the results and critically reviewed drafts and the final manuscript. CS contributed to the interpretation of the results and critically reviewed drafts and the final manuscript. JCF directed the overall project and contributed to the methodology design, data analysis and the final writing of the paper. All the authors read and approved the final manuscript

## Pre-publication history

The pre-publication history for this paper can be accessed here:

http://www.biomedcentral.com/1472-6947/10/68/prepub

## Supplementary Material

Additional file 1 **the correlation between cases of RSV (detected by laboratory means) and cases of bronchiolitis selected using ICD9**. pdf file with the graph depicting the correlation between cases of RSV (detected by laboratory means) and cases of bronchiolitis selected using ICD9 codes related to RSV. Click here for file

Additional file 2 **Comprehensive list of models considered in this work**. An Excel (.xlsx) spread sheet with the comprehensive list of the weather variable used in each of the 255 models considered here along with the sensitivity and specificity in both training and test for both high peak and low peak seasons. Click here for file
