# Supplementary material for: Predicting the start week of respiratory syncytial virus outbreaks using real time weather variables
Source: BMC Med Inform Decis Mak. 2010 Nov 2;10:68. doi: 10.1186/1472-6947-10-68 (PMC2987968; doi:10.1186/1472-6947-10-68)

Supplementary Material Figure: Comparison between the number of cases detected using the RSV laboratory results with those indentified using a generic ICD9 code for bronchiolitis. The comparison is shown for the years 2004 to 2008 for which both data are available.

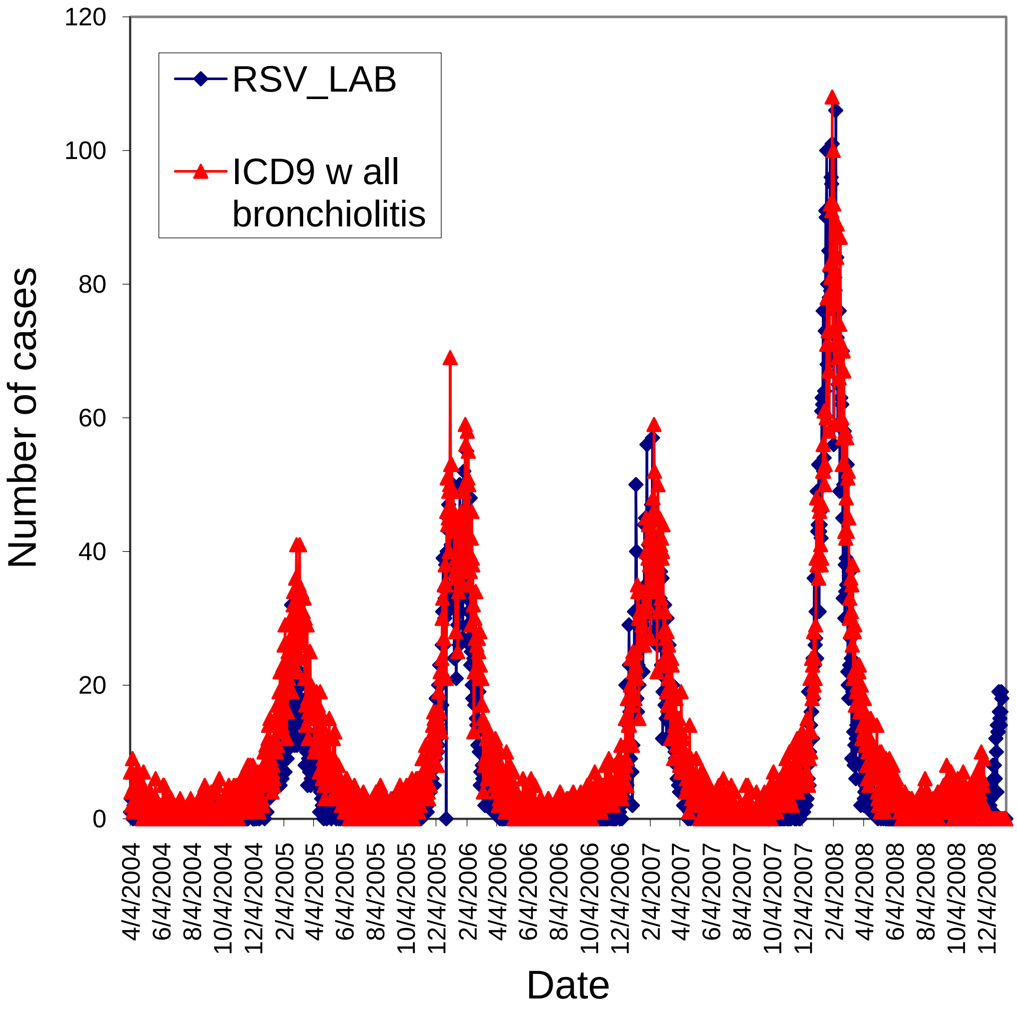

Supplement: Additional file 1 — the correlation between cases of RSV (detected by laboratory means) and cases of bronchiolitis selected using ICD9. pdf file with the graph depicting the correlation between cases of RSV (detected by laboratory means) and cases of bronchiolitis selected using ICD9 codes related to RSV. [file 1472-6947-10-68-S1.PDF]
